# Supplementary material for: Multiplexed Data-Independent Acquisition (mDIA) to Profile Extracellular Vesicle Proteomes
Source: Mol Cell Proteomics. 2026 Jan 8;25(2):101507. doi: 10.1016/j.mcpro.2026.101507 (PMC12887797; doi:10.1016/j.mcpro.2026.101507)
Supplement: Supplemental figures and tables [file mmc8.docx]

**Multiplexed Data-Independent Acquisition (mDIA) to Profile Extracellular Vesicle Proteomes**

Yi-Kai Liu^1^, Nathaniel Miller^1^, Marco Hadisurya^1^, Zheng Zhang^1^, and W. Andy Tao^1,2,3,4*^

^1^Department of Biochemistry, Purdue University, West Lafayette, IN 47907, USA

^2^Department of Chemistry, Purdue University, West Lafayette, IN 47907, USA

^3^Purdue Institute for Cancer Research, Purdue University, West Lafayette, IN 47907, USA

^4^Tymora Analytical Operations, West Lafayette, West Lafayette, IN 47906, USA

*Corresponding authors: taow@purdue.edu

**Supplemental Data**

Table S1. Variable window sizes for the optimal dia-PASEF method used in the benchmarking experiment (CCLP, EVs).

Table S2. Variable window sizes for the optimal dia-PASEF method used in the application experiment (CCLP+RBE+AG120, EVs).

Table S3. Variable window sizes for the optimal dia-PASEF method used in the application experiment (CCLP+RBE+AG120, cells).

Table S4. DDA-based spectral libraries generated from fractions.

Table S5. Evaluation of optimized dia-PASEF window placement using spectral libraries.

Figure S1. Optimal dia‐PASEF acquisition schemes for dimethyl labeling based mDIA.

Figure S2. Gene Ontology (GO) cellular component enrichment analysis of proteins identified from EV samples.

Figure S3. Dimethyl labeling efficiency.

Figure S4. Quantitative performance for the top 100 EV proteins.

Figure S5. Comparison of label-free DIA and mDIA using limited EV amounts.

Figure S6. Comparison of label-free DIA and mDIA using low EV peptide input.

Figure S7. Comparison of quantification precision between library-based and library-free pipelines in the EV-based application experiment.

Figure S8. Protein identification numbers from the cell-based application experiment using different mDIA pipelines.

Figure S9. Significantly regulated proteins in cell- and EV-based experiments.

Figure S10. EV protein candidates for identifying IDH1 mutant iCCA subtype and monitoring AG-120 therapeutic response.

**Supplemental Table S1. Variable window sizes for the optimal dia-PASEF method used in the benchmarking experiment (CCLP, EVs).**

| MS Type | Cycle Id | 1/K0 Begin [Vs/cm2] | 1/K0 End [Vs/cm2] | Start Mass [m/z] | End Mass [m/z] | CE [eV] |
| --- | --- | --- | --- | --- | --- | --- |
| MS1 | 0 | - | - | - | - | - |
| PASEF | 1 | 0.85 | 1.3 | 624.84 | 646.71 | - |
| PASEF | 1 | 0.7 | 0.85 | 300.19 | 390.93 | - |
| PASEF | 2 | 0.88 | 1.3 | 646.71 | 669.09 | - |
| PASEF | 2 | 0.7 | 0.88 | 390.93 | 423.62 | - |
| PASEF | 3 | 0.89 | 1.3 | 669.09 | 693.89 | - |
| PASEF | 3 | 0.7 | 0.89 | 423.62 | 449.29 | - |
| PASEF | 4 | 0.9 | 1.3 | 693.89 | 720.37 | - |
| PASEF | 4 | 0.7 | 0.9 | 449.29 | 469.55 | - |
| PASEF | 5 | 0.92 | 1.3 | 720.37 | 747.4 | - |
| PASEF | 5 | 0.7 | 0.92 | 469.55 | 488.74 | - |
| PASEF | 6 | 0.93 | 1.3 | 747.4 | 777.39 | - |
| PASEF | 6 | 0.7 | 0.93 | 488.74 | 507.95 | - |
| PASEF | 7 | 0.94 | 1.3 | 777.39 | 808.46 | - |
| PASEF | 7 | 0.7 | 0.94 | 507.95 | 527.32 | - |
| PASEF | 8 | 0.96 | 1.3 | 808.46 | 846.47 | - |
| PASEF | 8 | 0.7 | 0.96 | 527.32 | 545.81 | - |
| PASEF | 9 | 0.98 | 1.3 | 846.47 | 892.91 | - |
| PASEF | 9 | 0.7 | 0.98 | 545.81 | 564.83 | - |
| PASEF | 10 | 1 | 1.3 | 892.91 | 956.06 | - |
| PASEF | 10 | 0.7 | 1 | 564.83 | 584.55 | - |
| PASEF | 11 | 1.03 | 1.3 | 956.06 | 1045.6 | - |
| PASEF | 11 | 0.7 | 1.03 | 584.55 | 603.83 | - |
| PASEF | 12 | 1.15 | 1.3 | 1045.6 | 1444.23 | - |
| PASEF | 12 | 0.7 | 1.15 | 603.83 | 624.84 | - |

**Supplemental Table S2. Variable window sizes for the optimal dia-PASEF method used in the application experiment (CCLP+RBE+AG120, EVs).**

| MS Type | Cycle Id | 1/K0 Begin [Vs/cm2] | 1/K0 End [Vs/cm2] | Start Mass [m/z] | End Mass [m/z] | CE [eV] |
| --- | --- | --- | --- | --- | --- | --- |
| MS1 | 0 | - | - | - | - | - |
| PASEF | 1 | 0.86 | 1.3 | 647.89 | 670.39 | - |
| PASEF | 1 | 0.7 | 0.86 | 300.52 | 395.55 | - |
| PASEF | 2 | 0.89 | 1.3 | 670.39 | 694.06 | - |
| PASEF | 2 | 0.7 | 0.89 | 395.55 | 433.92 | - |
| PASEF | 3 | 0.91 | 1.3 | 694.06 | 719.36 | - |
| PASEF | 3 | 0.7 | 0.91 | 433.92 | 461.79 | - |
| PASEF | 4 | 0.92 | 1.3 | 719.36 | 746.88 | - |
| PASEF | 4 | 0.7 | 0.92 | 461.79 | 485.5 | - |
| PASEF | 5 | 0.93 | 1.3 | 746.88 | 774.39 | - |
| PASEF | 5 | 0.7 | 0.93 | 485.5 | 506.28 | - |
| PASEF | 6 | 0.95 | 1.3 | 774.39 | 805.39 | - |
| PASEF | 6 | 0.7 | 0.95 | 506.28 | 526.27 | - |
| PASEF | 7 | 0.96 | 1.3 | 805.39 | 838.95 | - |
| PASEF | 7 | 0.7 | 0.96 | 526.27 | 545.95 | - |
| PASEF | 8 | 0.98 | 1.3 | 838.95 | 877.51 | - |
| PASEF | 8 | 0.7 | 0.98 | 545.95 | 565.82 | - |
| PASEF | 9 | 1 | 1.3 | 877.51 | 924.01 | - |
| PASEF | 9 | 0.7 | 1 | 565.82 | 585.6 | - |
| PASEF | 10 | 1.02 | 1.3 | 924.01 | 985.54 | - |
| PASEF | 10 | 0.7 | 1.02 | 585.6 | 605.35 | - |
| PASEF | 11 | 1.05 | 1.3 | 985.54 | 1070.57 | - |
| PASEF | 11 | 0.7 | 1.05 | 605.35 | 626.35 | - |
| PASEF | 12 | 1.15 | 1.3 | 1070.57 | 1398.93 | - |
| PASEF | 12 | 0.7 | 1.15 | 626.35 | 647.89 | - |

**Supplemental Table S3. Variable window sizes for the optimal dia-PASEF method used in the application experiment (CCLP+RBE+AG120, cells).**

| MS Type | Cycle Id | 1/K0 Begin [Vs/cm2] | 1/K0 End [Vs/cm2] | Start Mass [m/z] | End Mass [m/z] | CE [eV] |
| --- | --- | --- | --- | --- | --- | --- |
| MS1 | 0 | - | - | - | - | - |
| PASEF | 1 | 0.85 | 1.3 | 638.41 | 660.86 | - |
| PASEF | 1 | 0.7 | 0.85 | 300.84 | 381.55 | - |
| PASEF | 2 | 0.88 | 1.3 | 660.86 | 683.89 | - |
| PASEF | 2 | 0.7 | 0.88 | 381.55 | 418.9 | - |
| PASEF | 3 | 0.89 | 1.3 | 683.89 | 709.03 | - |
| PASEF | 3 | 0.7 | 0.89 | 418.9 | 447.73 | - |
| PASEF | 4 | 0.91 | 1.3 | 709.03 | 736.43 | - |
| PASEF | 4 | 0.7 | 0.91 | 447.73 | 471.25 | - |
| PASEF | 5 | 0.92 | 1.3 | 736.43 | 765 | - |
| PASEF | 5 | 0.7 | 0.92 | 471.25 | 492.94 | - |
| PASEF | 6 | 0.94 | 1.3 | 765 | 796.41 | - |
| PASEF | 6 | 0.7 | 0.94 | 492.94 | 513.81 | - |
| PASEF | 7 | 0.96 | 1.3 | 796.41 | 830.42 | - |
| PASEF | 7 | 0.7 | 0.96 | 513.81 | 534.29 | - |
| PASEF | 8 | 0.98 | 1.3 | 830.42 | 868.41 | - |
| PASEF | 8 | 0.7 | 0.98 | 534.29 | 555.32 | - |
| PASEF | 9 | 1 | 1.3 | 868.41 | 914.77 | - |
| PASEF | 9 | 0.7 | 1 | 555.32 | 575.82 | - |
| PASEF | 10 | 1.03 | 1.3 | 914.77 | 977.89 | - |
| PASEF | 10 | 0.7 | 1.03 | 575.82 | 595.86 | - |
| PASEF | 11 | 1.06 | 1.3 | 977.89 | 1063.98 | - |
| PASEF | 11 | 0.7 | 1.06 | 595.86 | 616.04 | - |
| PASEF | 12 | 1.2 | 1.3 | 1063.98 | 1398.93 | - |
| PASEF | 12 | 0.7 | 1.2 | 616.04 | 638.41 | - |

**Supplemental Table S4**. **DDA-based spectral libraries generated from fractions.**

| Libraries | No. of precursors | No. of  modified peptides | No. of  peptides | No. of  proteins |
| --- | --- | --- | --- | --- |
| EVs_Benchmarking (CCLP) | 31,467 | 27,126 | 26,003 | 6,119 |
| EVs_Application (CCLP+RBE+AG120) | 48,401 | 42,625 | 38,860 | 9,410 |
| Cells_Application (CCLP+RBE+AG120) | 46,054 | 38,095 | 35,732 | 7,487 |

**Supplemental Table S5**. **Evaluation of optimized dia-PASEF window placement using spectral libraries.**

| Evaluation parameters | EVs_Benchmarking (CCLP) | EVs_Application (CCLP+RBE+AG120) | Cells_Application (CCLP+RBE+AG120) |
| --- | --- | --- | --- |
| Precursors within m/z-range [%] | 99.98 | 99.96 | 99.95 |
| Smallest diaPASEF window | 18.49 | 19.68 | 20.04 |
| Biggest diaPASEF window | 398.63 | 328.36 | 334.95 |
| Average diaPASEF window size | 47.67 | 45.77 | 45.75 |
| All proteins covered | 99.90% | 99.90% | 100.00% |
| All precursors covered | 98.60% | 98.70% | 98.10% |
| All doubly charged precursors covered | 100.00% | 100.00% | 99.90% |
| All triply charged precursors covered | 96.20% | 95.40% | 94.90% |
| All quadruply charged precursors covered | 99.40% | 99.70% | 98.60% |
| All singly charged precursors covered | 100.00% | 100.00% | 100.00% |


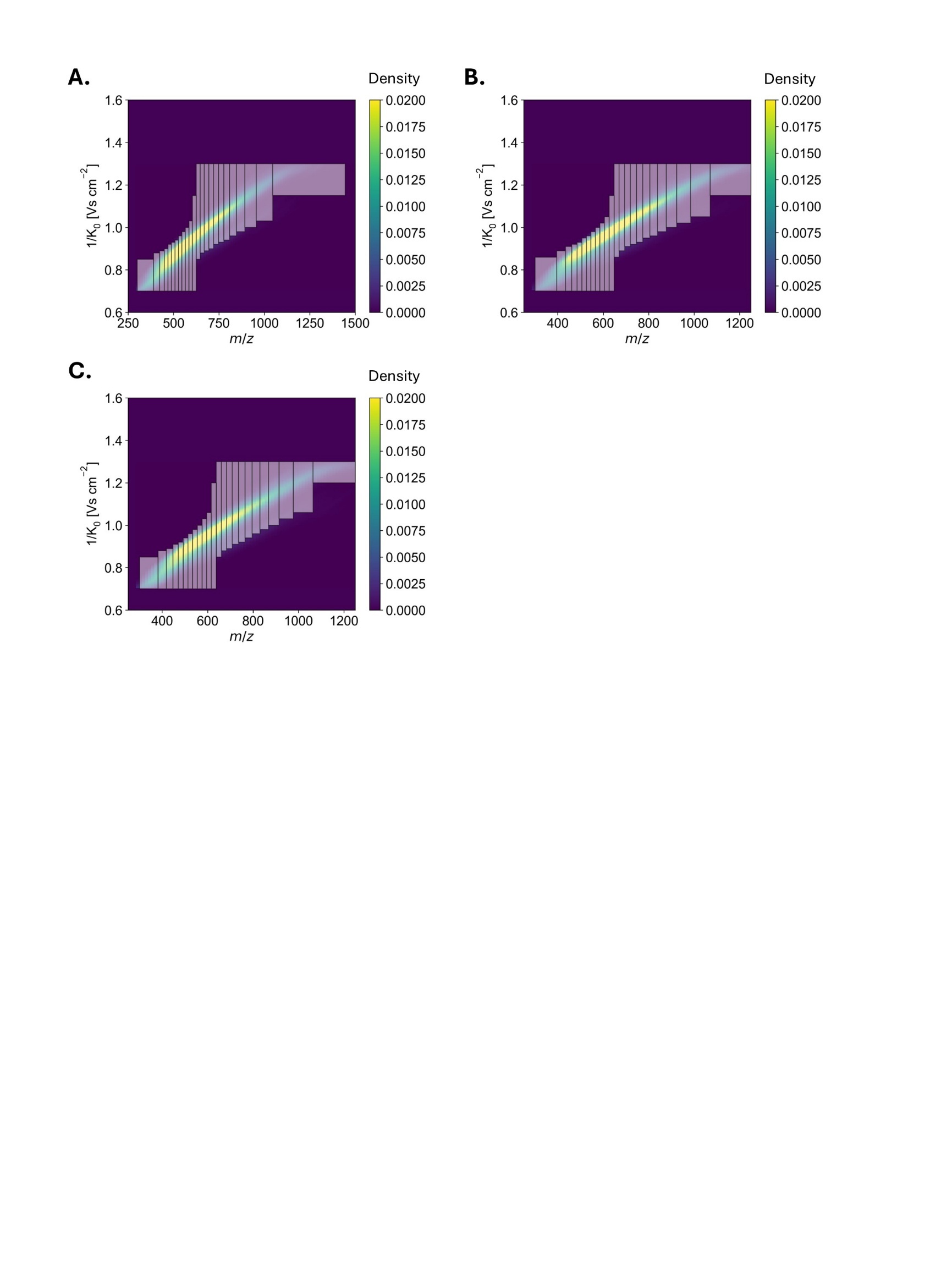


**Supplemental Figure S1**. **Optimal dia‐PASEF acquisition schemes for dimethyl labeling based mDIA.** A 12-scan dia-PASEF method was used for acquiring data from dimethyl-labeled peptides in (A) the benchmarking experiment (EVs), (B) the application experiment (EVs), and (C) the application experiment (cells). Each scheme consists of one MS1 scan followed by 12 dia-PASEF scans with variable m/z isolation widths and two ion mobility windows within a single cycle. The color scale represents the density of precursors across the m/z and 1/K₀ space.


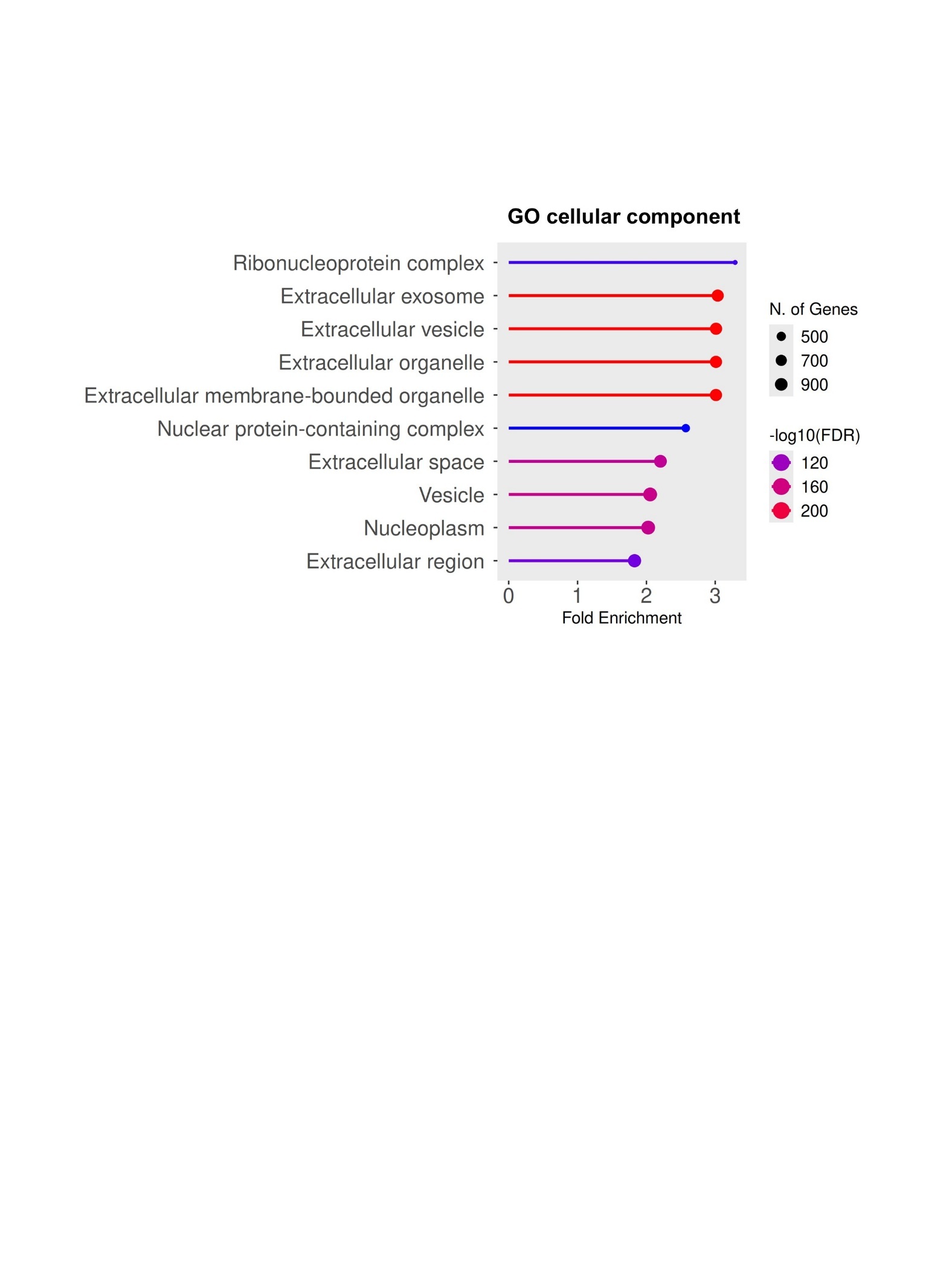


**Supplemental Figure S2. Gene Ontology (GO) cellular component enrichment analysis of proteins identified from EV samples.** GO enrichment analysis of the identified EV proteins from label-free proteomics revealed multiple EV-related cellular component terms. The size of each dot indicates the number of genes associated with each GO term, and the color intensity indicates statistical significance (–log10[FDR]).


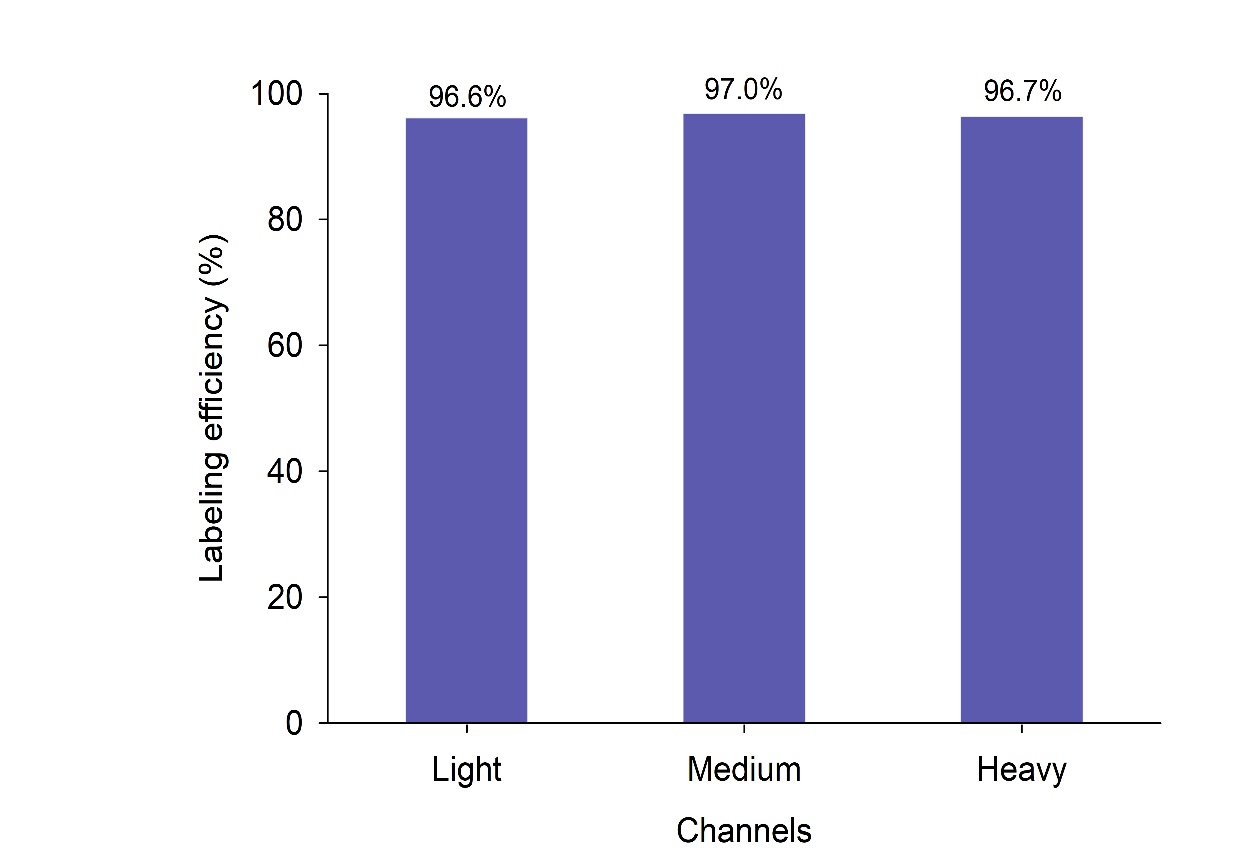


**Supplemental Figure S3. Dimethyl labeling efficiency.** Equal amounts of EV peptides were loaded into each dimethyl channel. The pooled sample was analyzed using DDA-PASEF and searched with FragPipe. Labeling efficiency was calculated based on the ratios of labeled peptides relative to all detected peptides (n = 1).


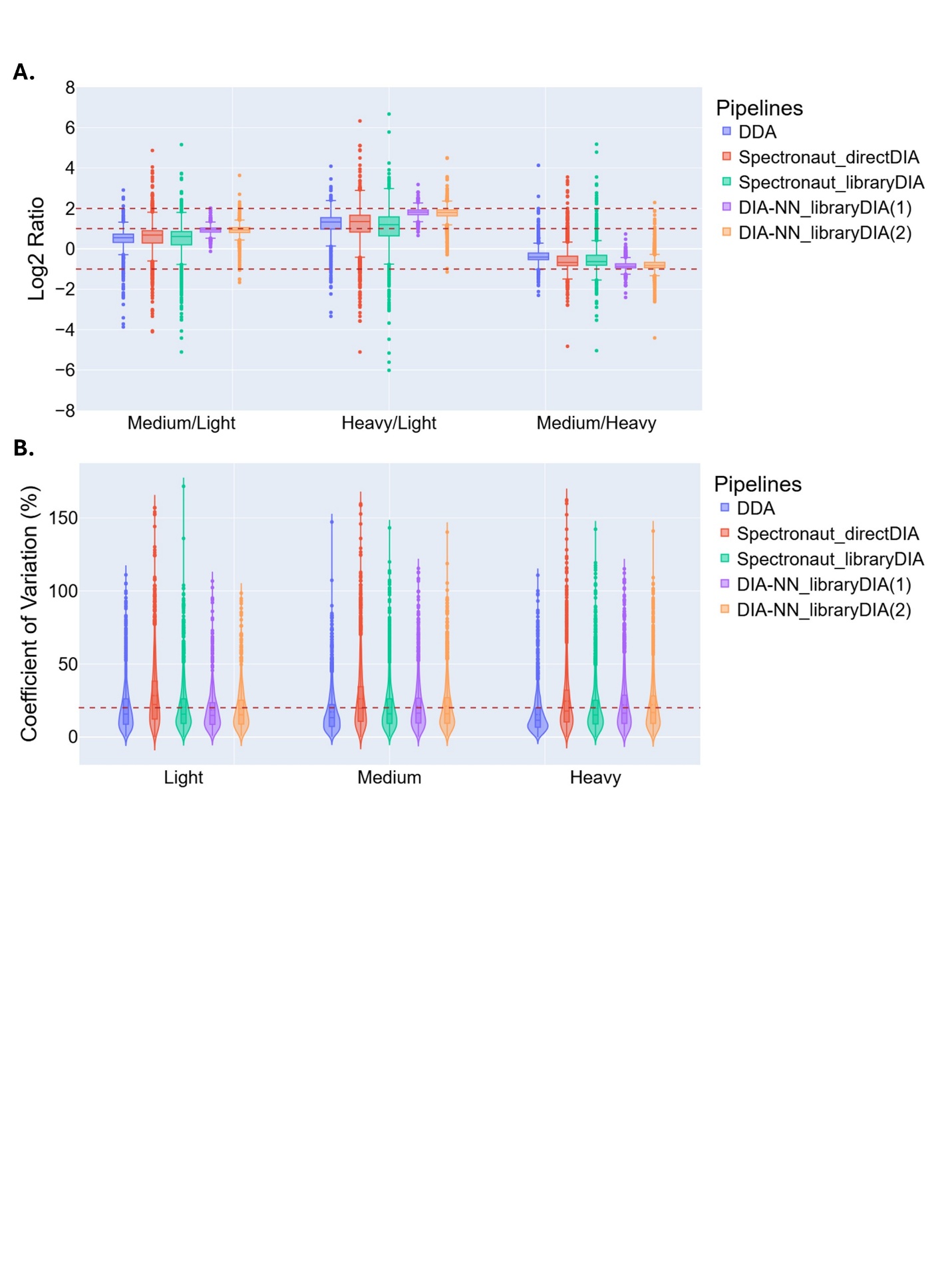


**Supplemental Figure S4. Quantitative performance for the top 100 EV proteins.** A, boxplots of log₂-transformed precursor intensity ratios for medium/light, heavy/light, and medium/heavy comparisons across pipelines. The dashed red lines indicate the expected log₂ ratios. B, CV distribution for precursors quantified across different pipelines and labeling channels. Violin plots display the spread of CV%, with a red dashed line indicating a 20% CV threshold. (1) project-specific fraction-based library. (2) predicted library from Thielert et al.


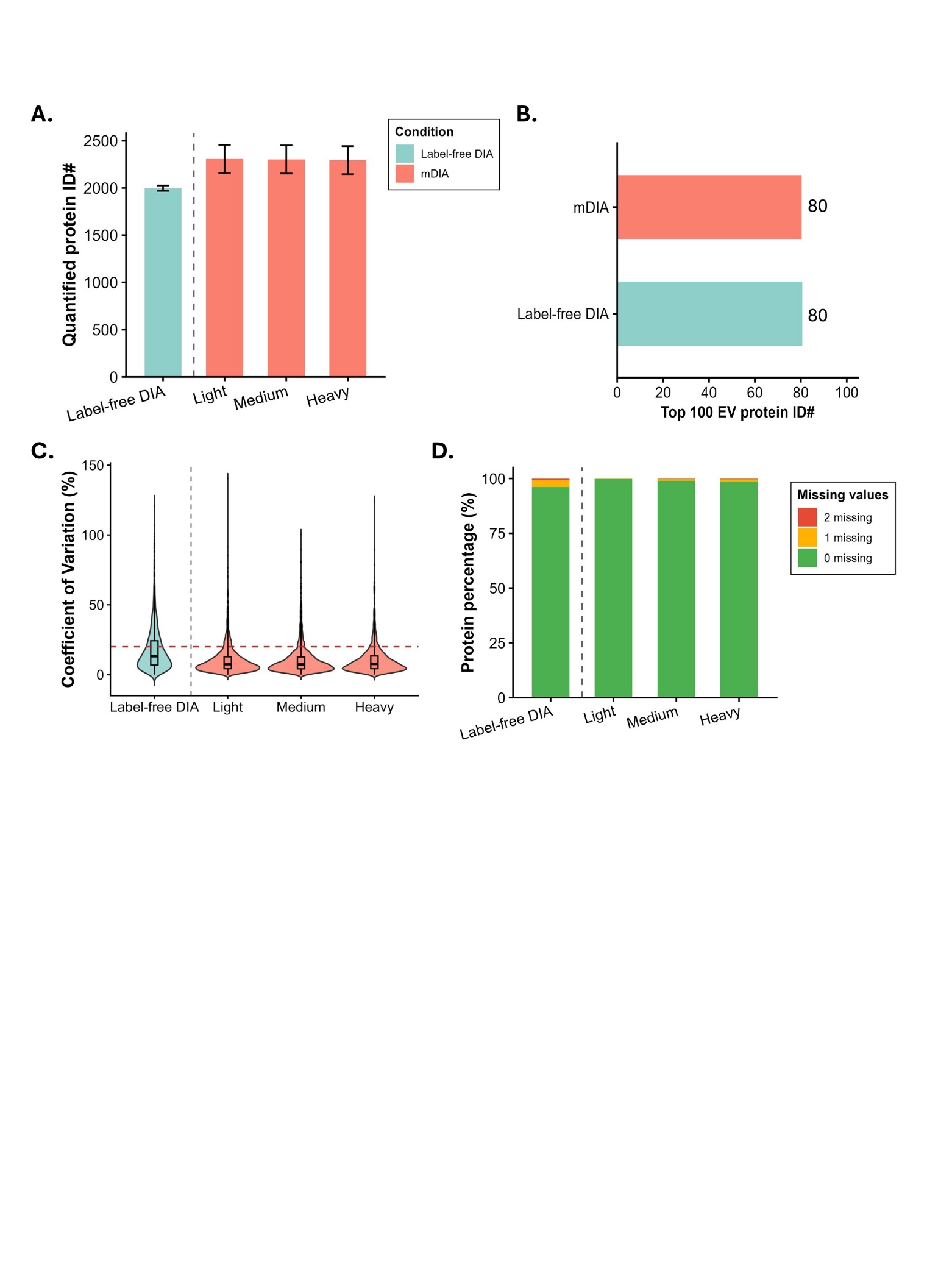


**Supplemental Figure S5. Comparison of label-free DIA and mDIA using limited EV amounts.** EV proteomic analyses were performed using label-free DIA and mDIA with EVs isolated from 50 µL of conditioned media. A, number of quantified protein identifications from label-free DIA and mDIA (light, medium, heavy channels). Error bars represent standard deviation across triplicates. B, the number of proteins from the top 100 most common EV proteins in the ExoCarta database identified by each method. C, CV distribution for protein quantified in different methods and labeling channels. Violin plots display the spread of CV%, with a red dashed line indicating a 20% CV threshold. D, percentage of proteins with 0, 1, or 2 missing values among triplicate analyses in label-free DIA and mDIA.


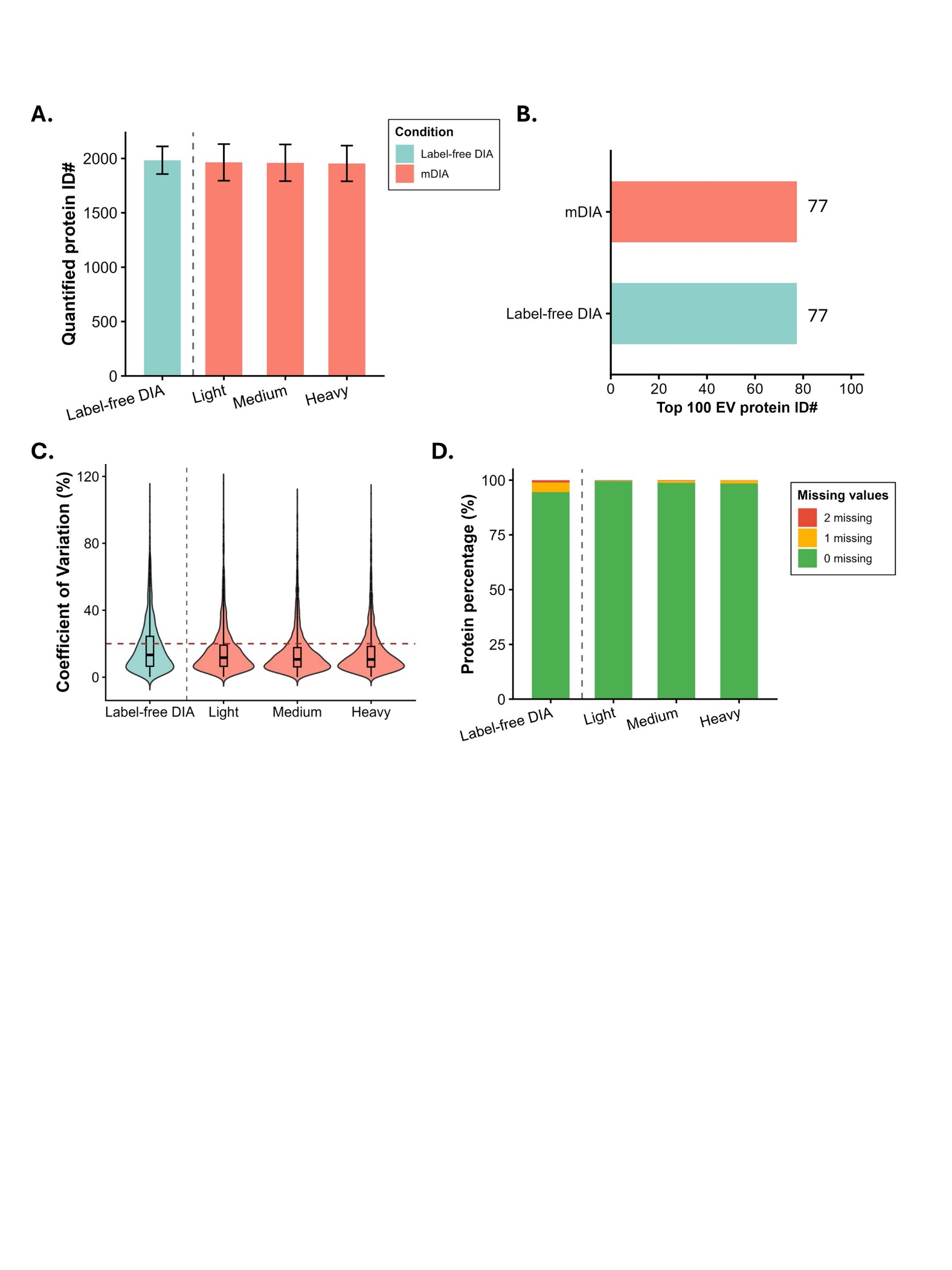


**Supplemental Figure S6. Comparison of label-free DIA and mDIA using low EV peptide input.** EV proteomic analyses were performed using label-free DIA and mDIA with 200 ng of digested EV peptides. A, number of quantified protein identifications from label-free DIA and mDIA (light, medium, heavy channels). Error bars represent standard deviation across triplicates. B, the number of proteins from the top 100 most common EV proteins in the ExoCarta database identified by each method. C, CV distribution for protein quantified in different methods and labeling channels. Violin plots display the spread of CV%, with a red dashed line indicating a 20% CV threshold. D, percentage of proteins with 0, 1, or 2 missing values among triplicate analyses in label-free DIA and mDIA.


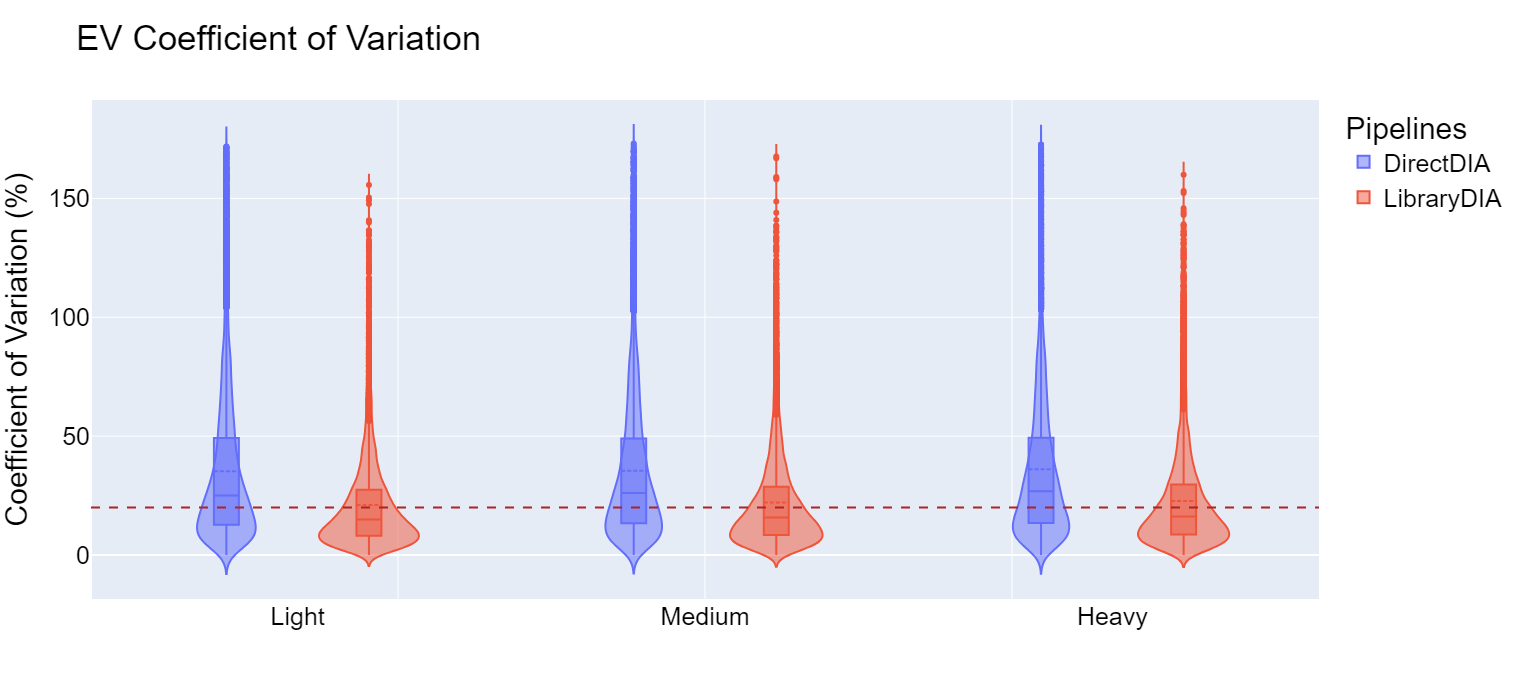


**Supplemental Figure S7**. **Comparison of quantification precision between library-based and library-free pipelines in the EV-based application experiment.** CV distributions for precursors quantified using the directDIA and library-based DIA pipelines in Spectronaut. Violin plots show the spread of CV%, with the red dashed line representing the 20% CV threshold.


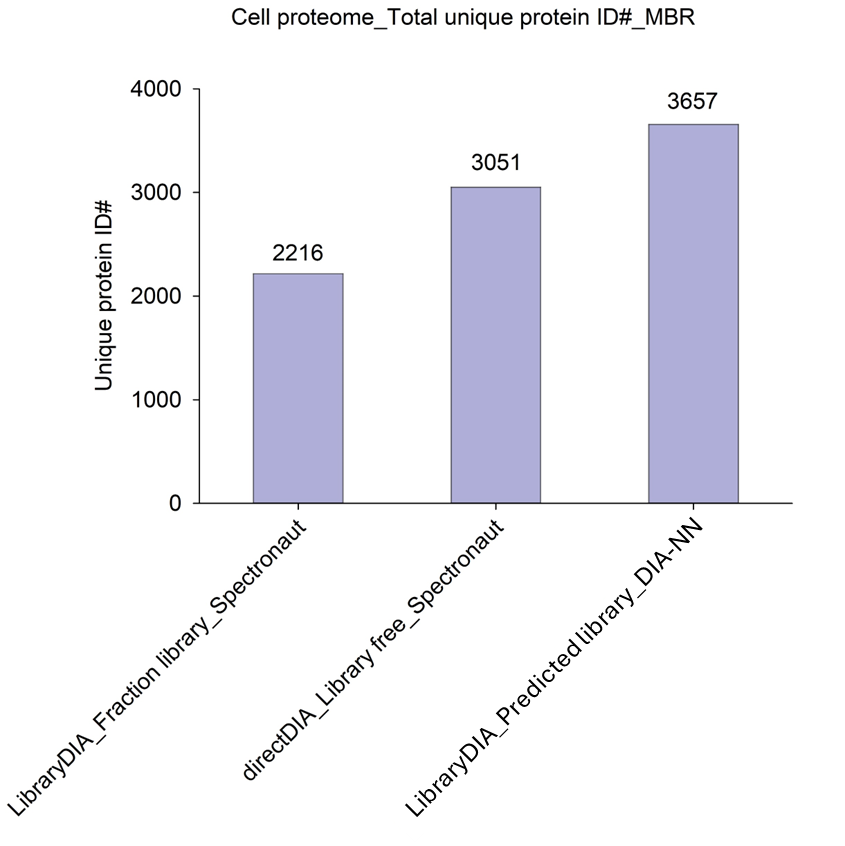


**Supplemental Figure S8**. **Protein identification numbers from the cell-based application experiment using different mDIA pipelines.** Each bar represents the total number of unique protein identifications across three channels in the match-between-runs (MBR) analysis (n=3). Fraction library is the project-specific library generated from fractions. Predicted library is from Thielert et al.


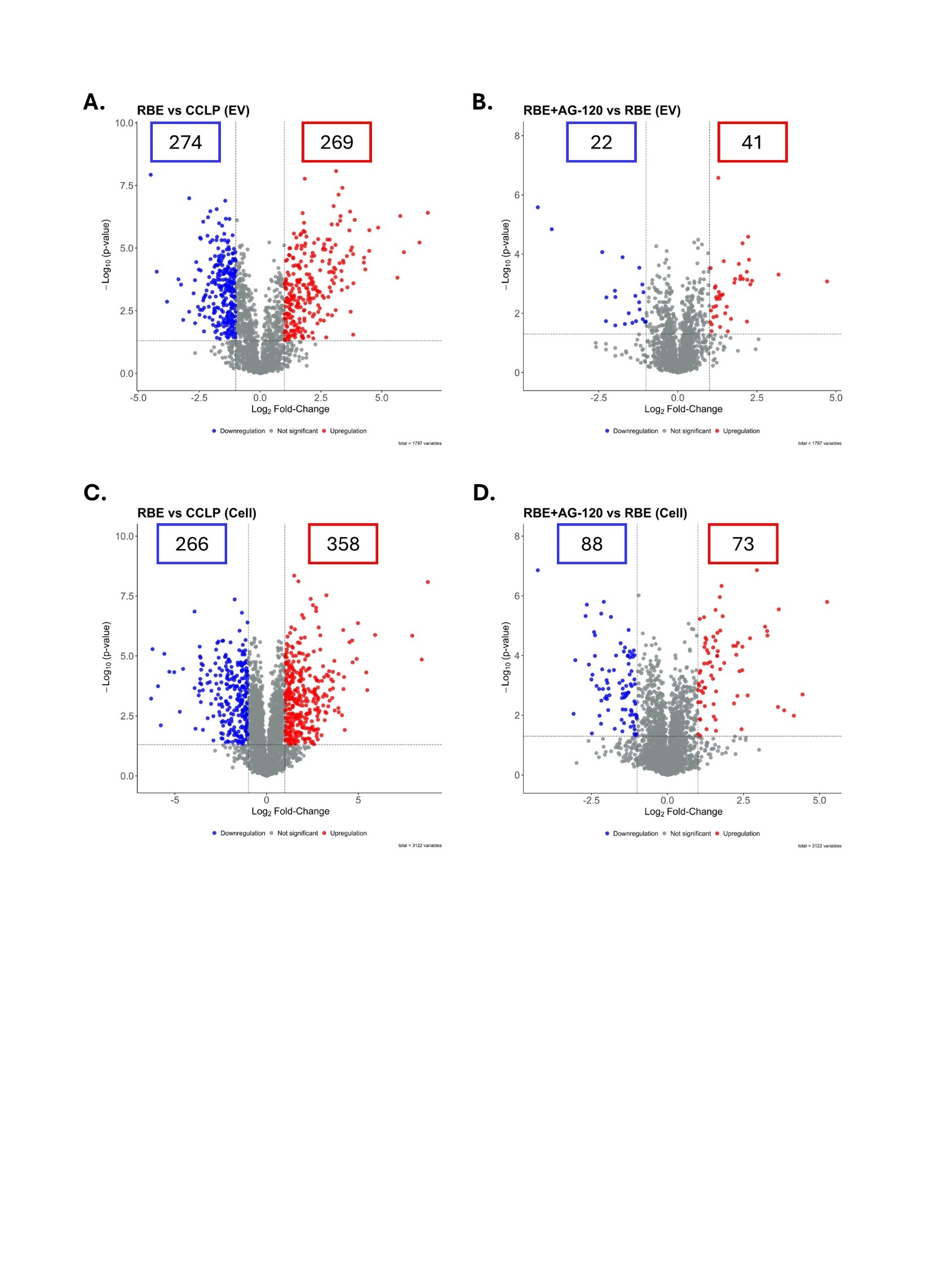


**Supplemental Figure S9. Significantly regulated proteins in cell- and EV-based experiments.** Volcano plots show comparisons of (A) RBE vs. CCLP in EVs, (B) RBE + AG-120 vs. RBE in EVs, (C) RBE vs. CCLP in cells, and (D) RBE + AG-120 vs. RBE in cells. Significance was determined using a two-sample Student's t-test with a p-value cutoff of 0.05 and an absolute log₂ fold-change cutoff of 1. The numbers of significantly up- and down-regulated proteins are highlighted.


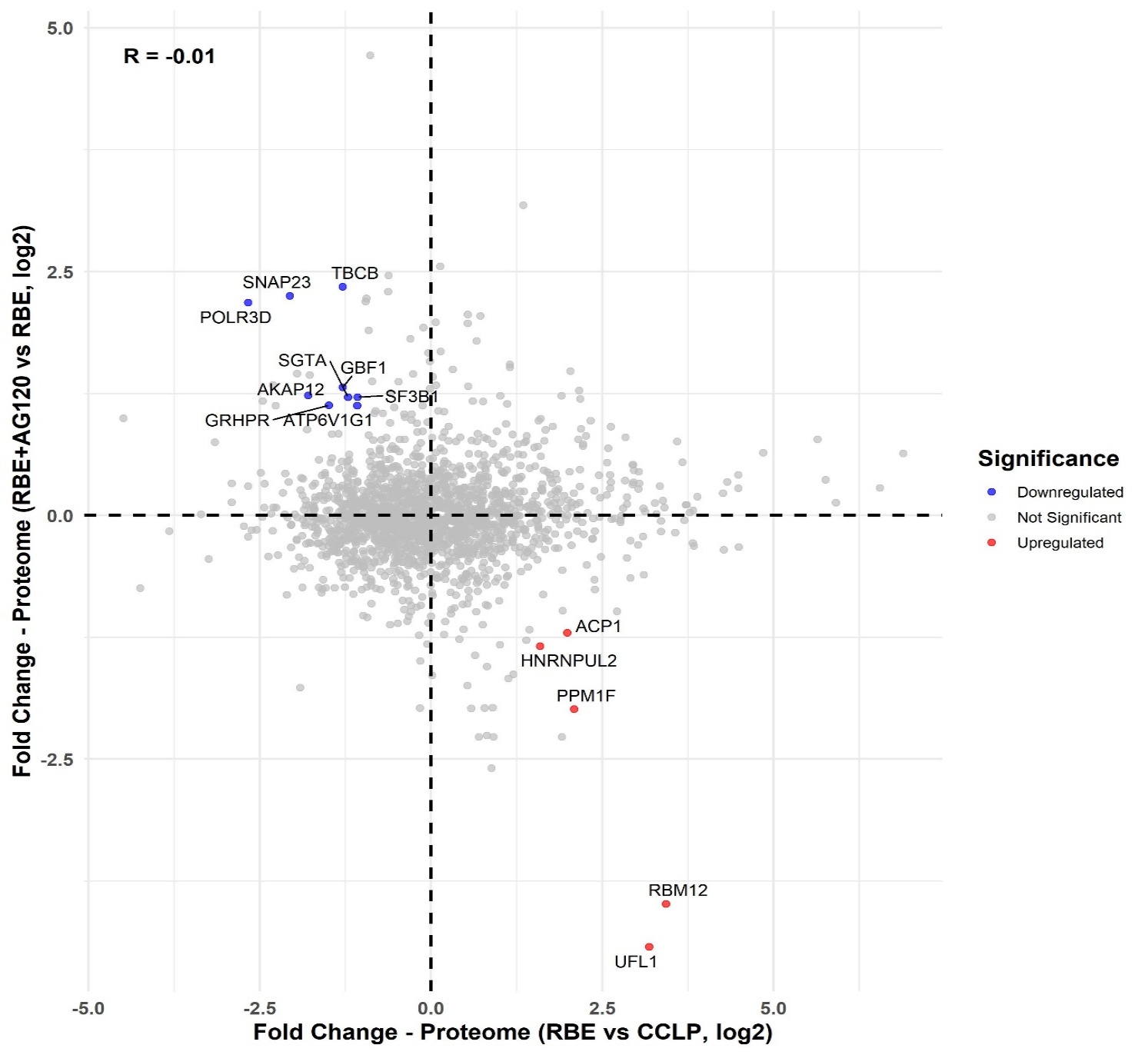


**Supplemental Figure S10.** **EV protein candidates for identifying IDH1 mutant iCCA subtype and monitoring AG-120 therapeutic response.** Scatter plot shows EV proteins with opposite expression patterns between IDH1 mutation and AG-120 treatment conditions. Proteins significantly upregulated in RBE vs. CCLP and significantly downregulated in RBE + AG-120 vs. RBE are shown in red, while proteins with the opposite pattern are shown in blue. Significance was determined using a two-sample Student's t-test with a p-value cutoff of 0.05 and an absolute log₂ fold-change cutoff of 1. All significant proteins are labeled.
